# Oxygen-evolution reaction by nickel/nickel oxide interface in the presence of ferrate(VI)

**DOI:** 10.1038/s41598-020-65674-x

**Published:** 2020-05-29

**Authors:** Mohammad Saleh Ali Akbari, Robabeh Bagheri, Zhenlun Song, Mohammad Mahdi Najafpour

**Affiliations:** 1 Department of Chemistry, Institute for Advanced Studies in Basic Sciences (IASBS), Zanjan, 45137-66731 Iran; 2 Key Laboratory of Marine Materials and Related Technologies, Zhejiang Key Laboratory of Marine Materials and Protective Technologies, Ningbo Institute of Materials Technology and Engineering, Chinese Academy of Sciences, Ningbo, 315201 China; 3 Center of Climate Change and Global Warming, Institute for Advanced Studies in Basic Sciences (IASBS), Zanjan, 45137-66731 Iran; 4 Research Center for Basic Sciences & Modern Technologies (RBST), Institute for Advanced Studies in Basic Sciences (IASBS), Zanjan, 45137-66731 Iran

**Keywords:** Chemistry, Catalysis, Heterogeneous catalysis

## Abstract

In this study, we investigate the effect of K_2_FeO_4_, as a new and soluble Fe salt at alkaline conditions, on oxygen-evolution reaction (OER) of Ni oxide. Both oxidation and reduction peaks for Ni in the presence and absence of Fe are linearly changed by (scan rate)^1/2^. Immediately after the interaction of [FeO_4_]^2-^ with the surface of the electrode, a significant increase in OER is observed. This could be indicative of the fact that either the [FeO_4_]^2-^ on the surface of Ni oxide is directly involved in OER, or, it is important to activate Ni oxide toward OER. Due to the change in the Ni(II)/(III) peak, it is hypothesized that Fe impurity in KOH or electrochemical cell has different effects at the potential range. At low potential, [FeO_4_]^2−^ is reduced on the surface of the electrode, and thus, is significantly adsorbed on the electrode. Finally, oxygen-evolution measurements of K_2_FeO_4_ and Ni_2_O_3_ are investigated under chemical conditions. K_2_FeO_4_ is not stable in the presence of Ni(II) oxide, and OER is observed in a KOH solution (pH ≈ 13).

## Introduction

Rapid depletion of fossil fuels and environmental problems have forced humans to find clean and sustainable energy sources^1^. Water-splitting reaction is a promising strategy for storing energy^2^. One limitation of water-splitting reaction is OER^3^. Thus, finding an efficient, stable, low-cost, earth-abundant, and environmentally friendly catalyst for this reaction is a challenging issue^4–8^.

Thomas A. Edison was the first to recognize the effect of Fe on decreasing the electrochemical capacity of Ni oxide. Following this finding, evidence from studies pointed to the fact that Fe ions significantly decrease the onset of OER of Ni oxide^9,10^. Ni oxides are poor catalysts for OER, but the incorporation of Fe into Ni oxide forms an efficient catalyst for OER^9–12^. Corrigan (1987) studied the effects of Fe (0.01% Fe present) on OER by Ni oxide^11^. The findings suggested that the incorporation of Fe ions in Ni oxide causes the Tafel slopes to drop from about 70 mV/decade to about 25 mV/decade^11^.

Boettcher’s group extensively investigated Fe-Ni oxide in OER^12^. The research group found that the increased conductivity by Fe ions in Ni oxide structure is not enough to explain the increased activity in Ni oxide toward OER^12^. They suggested that Fe affects the electronic structure of Ni oxide by inducing partial-charge transfer^12^. Their work also disproved the long-held view that β-NiOOH is intrinsically more active than γ-NiOOH, which is related to the increase in OER through the incorporation of Fe ions in Ni oxide^12^.

The Pourbaix diagram shows that Fe(VI), and Ni(IV) species could be formed under OER conditions^13,14^. Therefore, the oxidation states of Ni and Fe, and the mechanism of OER by Fe-Ni oxide are challenging issues in the field. The role of Fe on Ni oxide during OER has become a source of controversy.

Some experimental methods and DFT calculations of Ni-Fe oxides have shown that OER occurs at Fe-sites embedded in the Ni oxide^15^. Another study confirmed that OER could proceed on Ni(IV) sites^16,17^. Bard’s group suggested that Ni(IV) is a poor active site during OER and that the highly active sites are the dispersed Fe ions in the Ni-oxide matrix^18^.

Using XANES, Dau’s group detected Ni(IV)^19^. Jin, Alp, and Stah detected Fe(IV) by the operando Mössbauer spectroscopic studies of a 3:1 Ni:Fe layered hydroxide^20^. However, Fe(IV) ions do not account for the catalytic activity^20^. In 2018, Chorkendorff’s group electrochemically oxidized the nanoparticles of metallic Ni-Fe alloy in an electrolyte containing H_2_^16^O to form NiFe^16^O_*x*_H_*y*_; in the next step, they added H_2_^18^O to the electrolyte. No participation of lattice oxygen in NiFe^16^O_x_H_y_ was observed^21,22^.

Using ^18^O-labeling experiments in combination with *in situ* Raman spectroscopy, it was found that lattice oxygen is involved in OER by Ni and Ni/Co LDHs, but not when Ni-Fe and Ni/Co/Fe LDHs are involved^23^.

The roles of dissolved Ni species in OER was reported by He’s group^24^. Other elements do not considerably affect OER of Ni oxide. However, some groups reported that Fe ions in electrolyte react with the surface of Co, Ni, Cu, Ag, Au, but not with Ti electrodes, and form the active sites for OER^12,14^.

It was reported that nickel(II) and cobalt(II) have a significant effect on the decomposition of [FeO_4_]^2−^ ^25^.

Herein, OER by Ni oxide in the presence of [FeO_4_]^2−^ is reported. In addition to the electrochemical experiments, OER by [FeO_4_]^2−^ and Ni_2_O_3_ was also investigated, and the results are reported. These findings showed that in addition to the hypothesis of the effect of Fe on the oxygen-evolving activity of Ni oxide, the effect of Ni ions on the oxygen-evolving activity of high-valent Fe active sites should also be investigated.

## Results

In this study, [FeO_4_]^2−^ was used as an Fe salt, and its effect on Ni oxide was investigated during OER. Using [FeO_4_]^2−^ as an Fe salt has some advantages:

(i) It is a new Fe salt for investigating the effect of Fe on OER by nickel oxide.
(ii) In contrast to many Fe salts, [FeO_4_]^2−^ is soluble under alkaline conditions. Thus, it could be easily added to nickel oxide under the potential to allow direct observation of the effect of Fe on OER of nickel oxide.
(iii) In contrast to Fe(III), [FeO_4_]^2−^ has a negative charge, and its migration toward the anode is easier than the migration of Fe(OH_2_)_6_^3+^ with the positive charge.
(iv) In the absence of the potential or a reductant, it has relatively good stability at high pH (>12).
(v) It shows a sharp peak in the visible spectra (violet color), and thus, its concentration could be detectable.
(xvi) [FeO_4_]^2−^ has been proposed as an intermediate in OER^26^.
(xvii) By using [FeO_4_]^2−^, relatively homogeneous layers of Fe oxide on the Ni foam is obtained, which is a promising synthetic method.

The sodium or potassium, but not barium ferrate (VI), [FeO_4_]^2−^, is soluble in water^27^. [FeO_4_]^2−^, a tetrahedral ion with violet color, has a strong oxidizing activity and is relatively stable at high pH. [FeO_4_]^2−^ salt is produced by the oxidation of Fe in an aqueous medium under the alkaline conditions in the presence of Cl_2_, and by heating Fe filings in the presence of potassium nitrate. [FeO_4_]^2−^ has a reduction potential of +2.2 V to +0.7 V versus NHE at pH 0 and 14, respectively^27^.

In this study, a nickel foam was used as the source of nickel oxide. First, a Ni foam was placed under oxidation conditions at 10.0 V for 20 minutes in a two-electrode setup in a KOH solution (pH ≈ 13). Then it was used for the electrochemical investigation. Because soluble nickel compounds may affect the electrochemistry of the electrode^24^, no nickel oxide was used to purify^12^ the electrolyte in our experiments.

**Figure 1:**
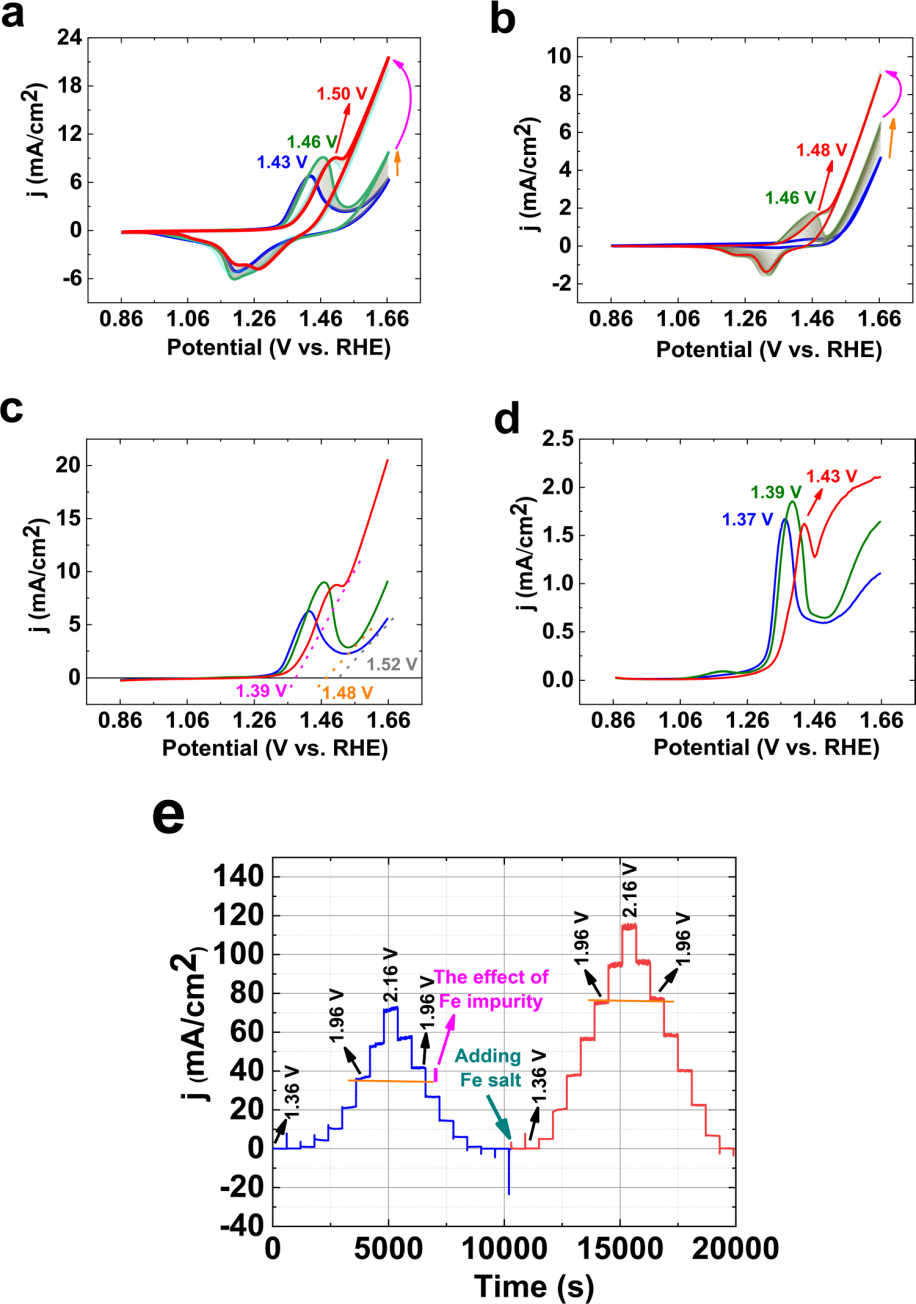
150 consecutive CVs of the Ni foam (**a**) or Ni foil (99.99% purity) (**b**) (scan rate: 25 mV/s) in the absence and presence of the Fe salt. After the 100^th^ CV, the Fe salt was added to the electrolyte. The 2^nd^ CV is shown in blue. The 150^th^ CV is shown in red. The orange arrow indicates an increase in OER in the presence of Fe impurity in KOH or electrochemical cell. The pink arrow indicates an increase in OER in the presence of the Fe salt. LSVs after 1^st^ and 100^th^ CVs for Ni foil before adding the Fe salt are shown in blue and green, respectively. LSV of Ni foil after adding the Fe salt (red). The numbers show the onsets of OER for LSVs (**c**). SWV of Ni foil before adding the Fe salt (blue). SWV of Ni foil after the 100 CVs (green) (**d**). SWV of Ni foil after adding the Fe salt and CV (red). The numbers show the peaks for Ni oxidation (**d**). Multi-step amperometry at 1.36–2.16 V before adding the Fe salt (blue) and after adding the Fe salt (red). The green arrow indicates the time of the addition of the Fe salt (**e**). The small orange line indicates the effect of Fe impurity on the current density at 1.96 V. KOH (pH ≈ 13) was used for all experiments.

LSV of the Ni foam in KOH (pH ≈ 13) showed that before adding [FeO_4_]^2−^, the onset of OER is observed at 1.52 V (in the paper, all potentials are reported vs. RHE) (Fig. 1a). Ni(II)/(III) oxidation is also revealed at 1.43 V (Eq. 1):

1 $${\rm{Ni}}{({\rm{OH}})}_{2}+{{\rm{OH}}}^{-}\to {\rm{NiOOH}}+{{\rm{e}}}^{-}+{{\rm{H}}}_{2}{\rm{O}}$$

The consecutive CVs are interesting in that they show the effect of the impurity of Fe and the addition of [FeO_4_]^2−^. As shown in Fig. 1a, in the absence of the [FeO_4_]^2-^ and the presence of the impurity of Fe impurity in KOH or electrochemical cell, an increase in OER (orange arrow) is observed during the consecutive CVs. The Ni oxidation peak shifts from 1.43 to 1.46 V during the consecutive CVs (Fig. 1a). After adding the Fe salt (the final concentration of Fe salt was 0.25 mM. If another concentration of Fe salt was used, it was written in the related captions), a significant change is observed in OER (pink arrow), and the Ni oxidation peak is observed at 1.50 V (Fig. 1a).

The same CV is observed for a pure metallic Ni foil (99.99% purity; Fig. 1b). For a pure metallic Ni foil, the first no peak is observed for Ni(II)/(III) oxidation, since no pre-treatment at high potential was performed for the foil. However, after a few consecutive CVs, the peak at 1.46 V was observed, which shows a shift to the higher potential at 100^th^ CV (1.48 V) (Fig. 1b). To find the effect of the amounts of Ni oxide on OER, a Ni foam without any anodization at 10.0 V was also investigated, which showed similar characteristics to the anodized Ni foil (Fig. S1).

After 100 consecutive CVs at a scan rate of 25 mV/s in the absence of adding Fe ions, the onset of OER and the peak are observed at 1.48 and 1.47 V by LSV, respectively (Fig. 1c). The changes correspond to the incorporation of Fe impurity in KOH or electrochemical cell to Ni oxide. After adding Fe salt, the onset of OER was observed at 1.39 V. The related peak to Ni(II)/(III) oxidation is observed at 1.50 V. The peak shape is significantly changed in the presence of adding Fe ions.

SWV indicated the peaks for Ni(II)/(III) oxidation before and after adding the Fe salt. Before adding the Fe salt in KOH (pH ≈ 13), a peak at 1.37 V was observed (Fig. 1d), which corresponded to Ni(II)/Ni(III) oxidation; after 50 consecutive CVs, this peak shifted to 1.39 V, which was related to the effect of impurity in KOH or electrochemical cell. After adding Fe, the peak was observed at 1.43 V (Fig. 1d). The multi-step amperometry at 1.36–2.16 V before adding the Fe salt (blue) and after adding the Fe salt (red) is shown in Fig. 1e. Before adding the Fe salt at 1.96 V, 5 mA increase in OER was observed, which was related to impurity in KOH or electrochemical cell (pink line).

**Figure 2:**
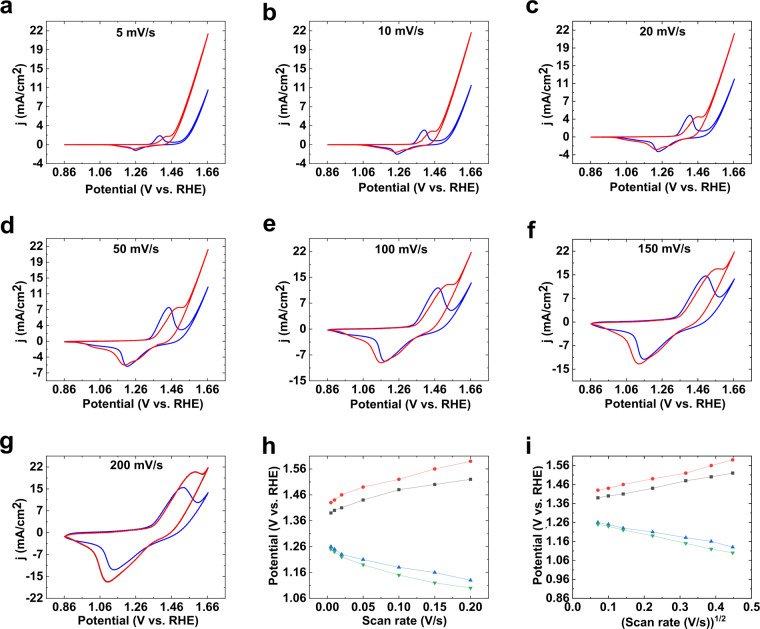
CVs of the Ni foam at different scan rates in the absence (blue) and presence of the Fe salt (red) (**a**–**g**). Potential vs. (scan rate) (**h**) and potential vs. (scan rate)^1/2^ (**i**) plots for Ni foam in the absence and presence of the Fe salt. KOH (pH ≈ 13) was used for all experiments. For (**h**,**i)**, red and black indicate the Ni(II)/(III) oxidation peaks in the presence and absence of the Fe salt, respectively; green and blue indicate the Ni(III)/(II) reduction peaks in the presence and absence of the Fe salt, respectively.

In the next step, the effect of different scan rates on OER, Ni oxidation and reduction peaks were investigated (Fig. 2). In all recorded scan rates, the Ni oxidation/reduction peaks in the absence and presence of Fe were observed. Both oxidation and reduction peaks for Ni in the presence and absence of Fe shifted linearly by (scan rate)^1/2^ (Fig. 2i), which showed freely diffusing species were important to form these peaks. The oxidation peaks shifted to higher potentials, and reduction peaks shifted to lower potential in the presence of Fe ions.

**Figure 3:**
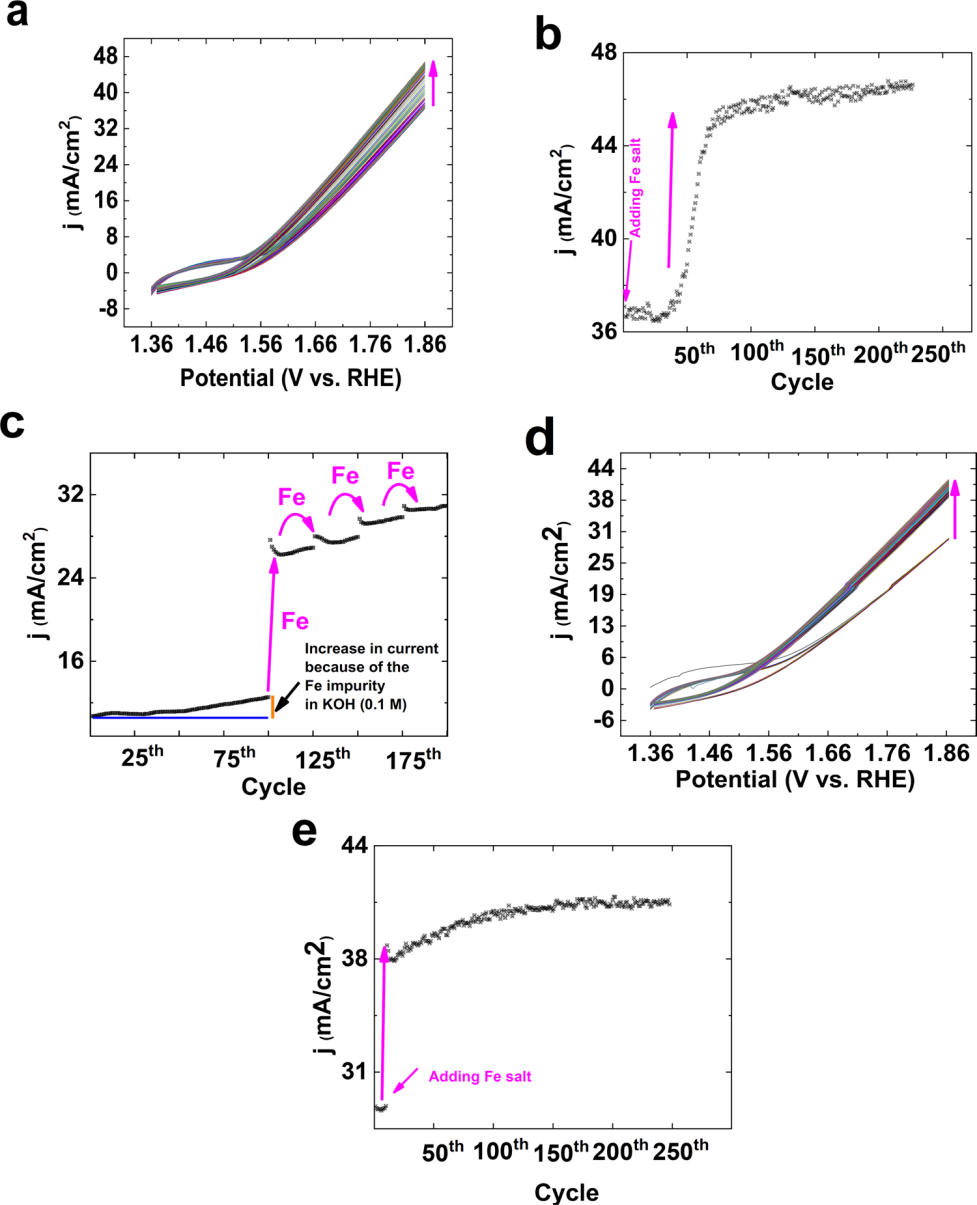
The effect of the Fe salt in the form of solid powder on CVs (scan rate: 25 mV/s) of Ni foam (**a**). The current density at 1.86 V for the consecutive CVs in the image **a** (**b**). The effect of continuous addition of Fe salt (final concentrations in each step: 0.25, 0.50, 0.75 and 1.0 mM) on the current density at 1.86 V for the consecutive CVs (KOH, pH ≈ 13) of Ni foam (**c**). The effect of large amounts of the Fe salt (final concentration: 12.6 mM) on CVs of Ni foam (scan rate: 25 mV/s, KOH, pH ≈ 13) (**d**). The current density at 1.86 V for the consecutive CVs in the image **d** (**e**).

In the next step, the Fe salt was added to the electrolyte in the form of solid powder, and CVs were recorded (Fig. 3a,b). The salt slowly diffused toward the electrode, and an improvement in OER was observed. Interestingly, immediately after the interaction of the Fe salt with the electrode, a significant increase in OER was observed (see the video, SI). This could show that Fe ions on the surface of Ni oxide were directly involved in OER or were important to the activation of Ni oxide toward OERs. After 80 consecutive CVs, a constant current was observed for OER (Fig. 3a,b), which showed that a few Fe sites were enough to optimize OER by Ni oxide.

The effect of the step by step addition of Fe ions to Ni oxide was studied. To inhibit the effect of the Fe salt on the cathode, the experiment was performed in a two-cell setup. As shown in Fig. 3c, at 1.46 V in KOH (pH ≈ 13), adding the Fe salt in the first step had a significant effect. However, adding more Fe salt had less effect on OER. If Ni sites are the active sites for OER, a decrease in OER may be observed after significantly covering the Ni sites by Fe ions. Surprisingly, however after adding more Fe salt, OER was increased. Thus, it is hypothesized that Fe sites could be active sites for OER.

The effect of large amounts of the Fe salt (12.6 mM) on OER of the Ni foam was investigated (Fig. 3d,e). Adding large amounts of Fe salt had no significant effect on OER compared to a small amount of Fe salt. After 100 consecutive CVs, a constant current was observed for the electrode in the presence of the Fe salt (Fig. 3d,e).

**Figure 4:**
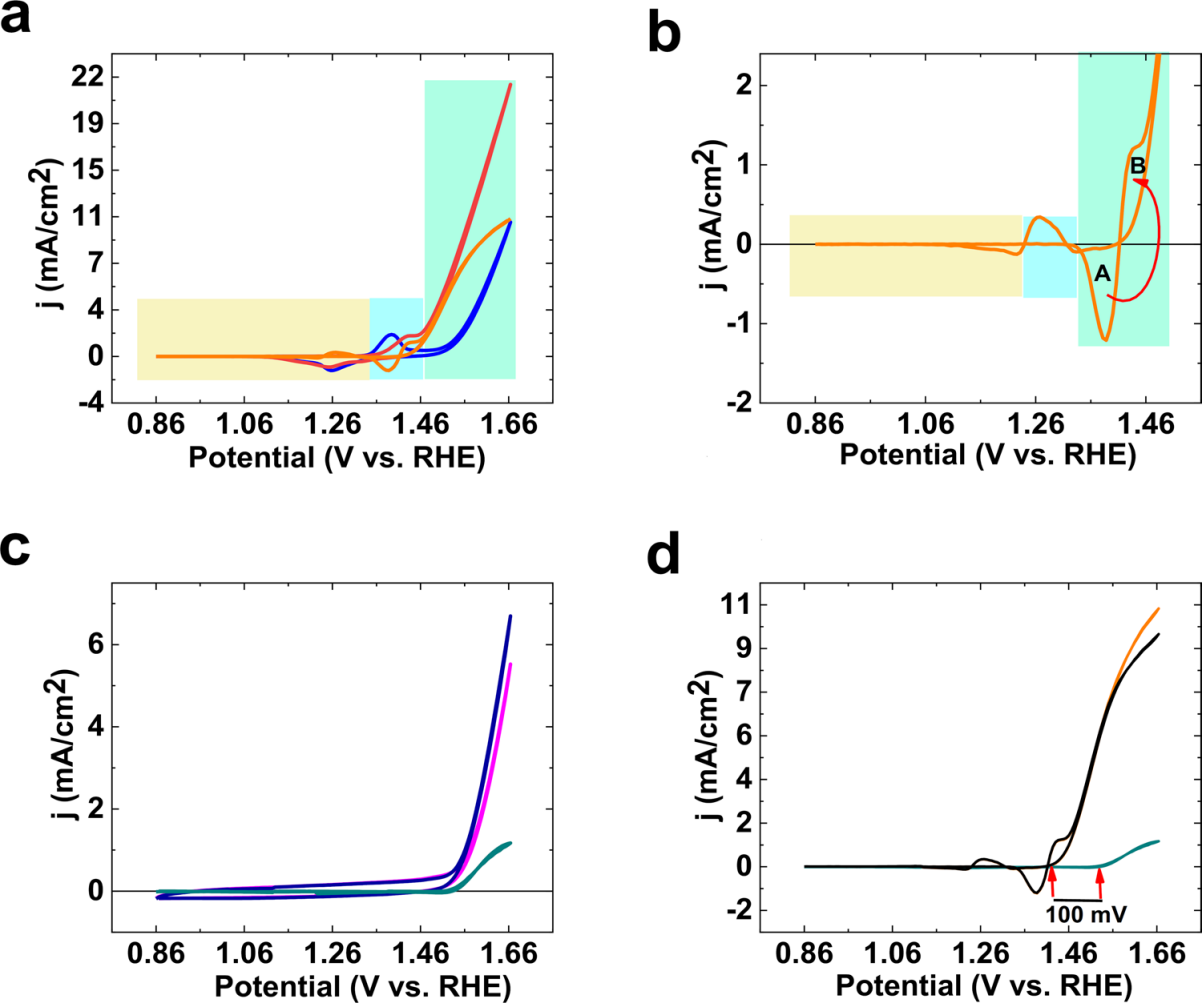
The subtraction (orange plot) of CV in the absence (blue CV) and presence (red CV) of the Fe salt (**a**,**b**). In the presence of the Fe salt (0.25 mM), the subtraction shows that the Fe salt decreases the capacitance of Ni oxide at 1.36–1.46 V range (A area). On the other hand, it seems that these charges are used by Fe to increase OER (B area). The subtraction (green plot) of CV in the absence (pink CV) and presence (blue CV) of the Fe salt in the presence of graphite (**c**). The subtraction of orange plot in (**b)** of green plot in **c** (**d**). All scan rates were 25 mV/s and KOH (pH ≈ 13).

An interesting idea is to analyze the effect of the Fe salt on the CV of Ni oxide. The subtraction (Fig. 4a, orange plot) of the CV in the absence of the Fe salt and the CV in the presence of the Fe salt is shown in Fig. 4b. It seems that the redox property of Ni(III) was used by Fe to increase in OER (B area in Fig. 4b). In other words, it is hypothesized that Ni sites, which remain at high oxidation states before the addition of Fe, are pushed to be discharged by Fe ions toward OER in the presence of Fe (B area in Fig. 4b).

**Figure 5:**
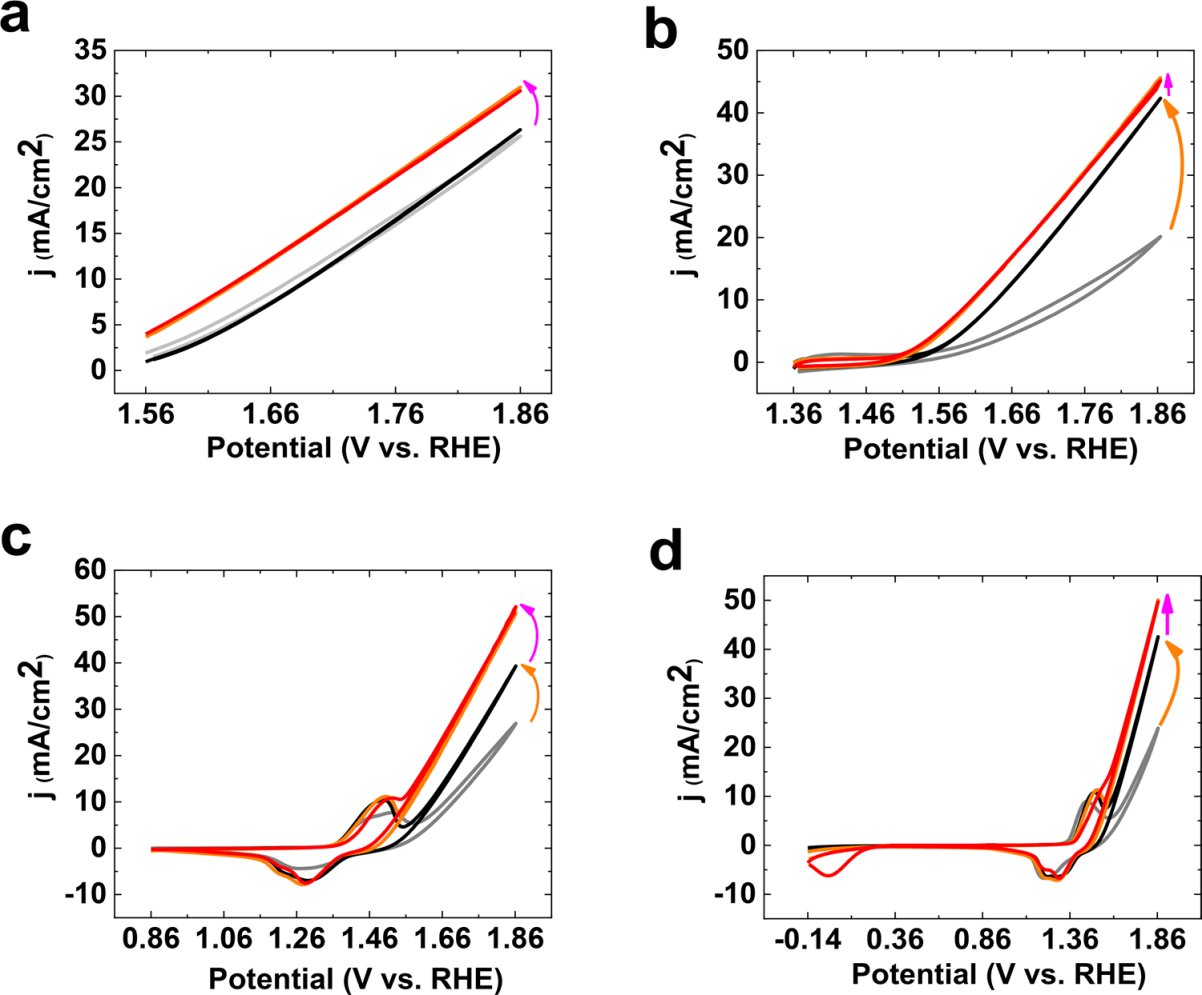
CV of the Ni foam at different potential ranges in the absence (grey), after the 50^th^ CV (black) and the presence of the Fe salt (1^st^ CV: orange; 50^th^ CV: red) (scan rate: 25 mV; KOH (pH ≈ 13)) (**a**–**d**).

Graphite electrode has no synergistic interaction with the Fe salt and the subtraction of the CV for graphite (green plot) in the absence and presence of Fe shows the effect of Fe on the electrode toward OER (Fig. 5c). It is interesting that the green and orange plots are comparable (Fig. 4b,c), if we withdraw the Ni(II)/(III) oxidation/reduction peaks. The orange plot is just bigger, and its onset of OER is observed at a low overpotential. The similarity between orange and green plots could show that in Fe-Ni oxide, similar to the case where Fe is on graphite, the Fe ions are active sites for OER.

If we subtract the pure effect of Fe (green plot) from the orange plot in Fig. 4d, the synergistic effect of Fe on nickel could be calculated (black plot). As shown in Fig. 4d, it is hypothesized that Ni(II)/(III) oxidation occurs before OER in pure Ni oxide and the absence of Fe. However, after adding the Fe salt, Ni(II)/(III) oxidation occurs in the same potential as the onset of OER. The onset of OER is observed at 100 mV lower by Fe-Ni oxide than Fe ions on graphite (Fig. 5d).

In the next study, the effect of the potential range on the effect of Fe on OER by Ni oxide was investigated (Fig. 5). Before adding the Fe salt at (1.56–1.86 V) range (Fig. 5a), the consecutive CVs showed no clear increase in OER. After adding the Fe salt at this range (Fig. 5a), an increase in OER (1.2 times) was observed, which was constant during the time of the reaction.

Before adding the Fe salt at (1.36–1.86 V) range, consecutive CVs showed an increase in OER (two times) at the 150^th^ CV (Fig. 5b). After adding the Fe salt at this range, a few increases (1.1 times) in OER was observed, which was constant during the time of reaction (Fig. 5b).

Before adding the Fe salt at (0.86–1.86 V) range (Fig. 5c), the consecutive CVs showed an increase in OER (1.5 times) at the 150^th^ CV. After adding the Fe salt at this range, a few increases (1.3 times) in OER was observed, which was constant during the time of reaction (Fig. 5c).

Before adding the Fe salt at (−0.14–1.86 V) range (Fig. 5d), the consecutive CVs showed an increase in OER (1.75 times) at the 150^th^ CV. After adding the Fe salt at this range, a few increases (1.15 times) in OER was observed, which was constant during the time of reaction (Fig. 5d).

Before adding the Fe salt, it seems that the activation factor needed a potential of less than 1.56 V. Given the change in the Ni(II)/(III) peak, it is hypothesized that impurity in KOH or electrochemical cell is the active factor. At a low potential, Fe ions are reduced on the surface of the electrode, and thus, are significantly adsorbed on the electrode. Thus, after the 150^th^ CV, a high activity for Ni oxide is observed. After adding Fe impurity on the surface of the electrode at a low potential, an Fe saturation occurs for Fe ion on the surface of the electrode, and the current remains constant. The related peak to Fe(II)/Fe(III) oxidation/reduction in Ni-Fe oxide is displayed at 0.36 V/ −0.06 V, respectively (Eq. 2):

2 $${\rm{Fe}}{({\rm{OH}})}_{2}+{{\rm{OH}}}^{-}\to {\rm{FeOOH}}+{{\rm{e}}}^{-}+{{\rm{H}}}_{2}{\rm{O}}$$

**Figure 6:**
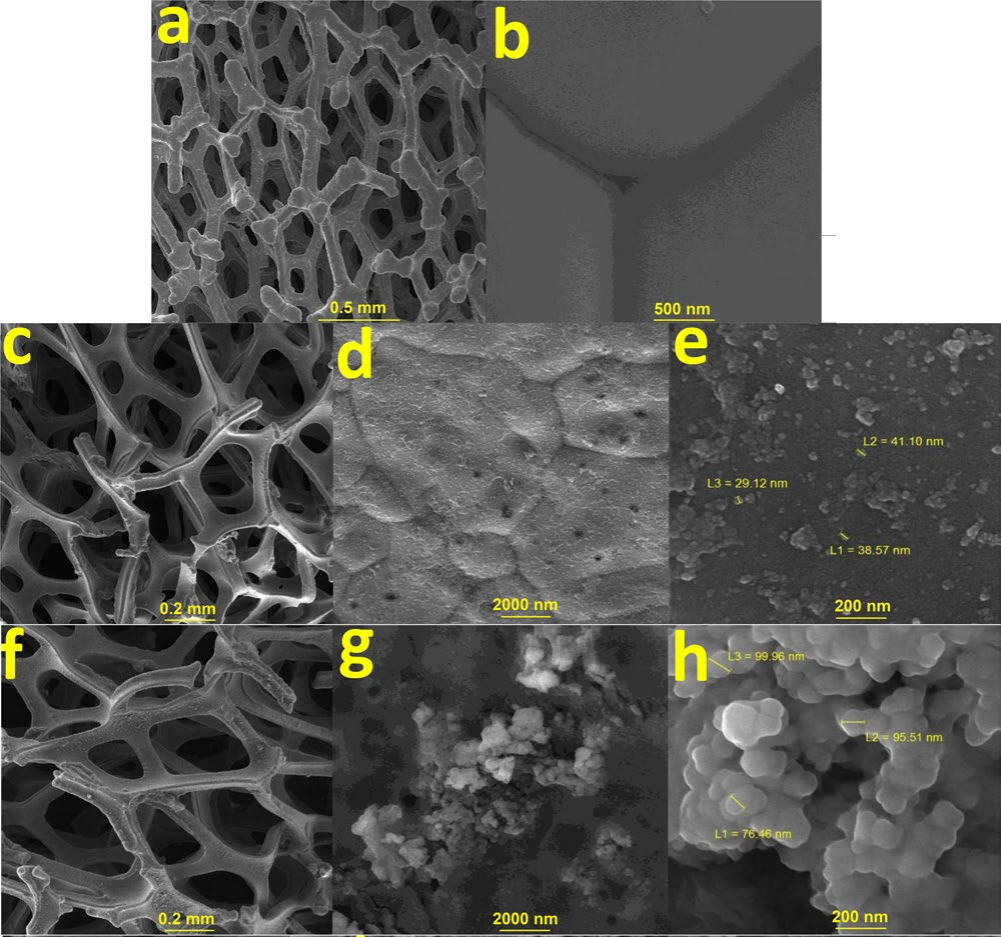
SEM images of the fresh Ni foam with different magnifications (**a**,**b**). SEM images of the operated Ni foam at 10.0 V with different magnifications (**c**–**e**). SEM images of the anodic Ni foam in the presence of the Fe salt with different magnifications (**f**–**h**). Amperometry at 1.46 V in KOH (pH ≈ 13) in a two-compartment electrochemical cell for 2 hours.

SEM images show that a fresh Ni foam has a cellular structure with high porosity and a large volume fraction of pores (Figs. 6a,b, S2). After operating the Ni foam at 10.0 V, a few corrosive points (ca. 10 nm) were observed on the surface of the cellular structure of the foam (Figs. 6c,d, Fig. S3 and S4). Small particles (20–40 nm) were also observed on the surface of the Ni foam (Fig. 6c,d). Under the operation at 10.0 V, no detectable iron was observed by EDX-SEM on the surface of the foam.

X-ray photoelectron spectroscopy (XPS) was used to characterize the operated foam at 10.0 V. Ni 2p peak showed significant split spin-orbit components (17.8 eV). Ni 2p3/2 spectrum of the operated foam at 10.0 V indicated a peak at 855.3 eV, which is related to Ni hydroxide (Fig. S5a)^28^. Ni 2p1/2 was also observed at 873 eV. Satellite features for Ni 2p3/2 and Ni 2p1/2 were displayed at 861 and 879 eV, respectively.

In addition, O 1 s component after the operation showed a peak at 531.1 attributed to oxygen in Ni hydroxide, OH, and OH_2_ groups on the surface of the electrode (Fig. S5b)^29^.

**Figure 7:**
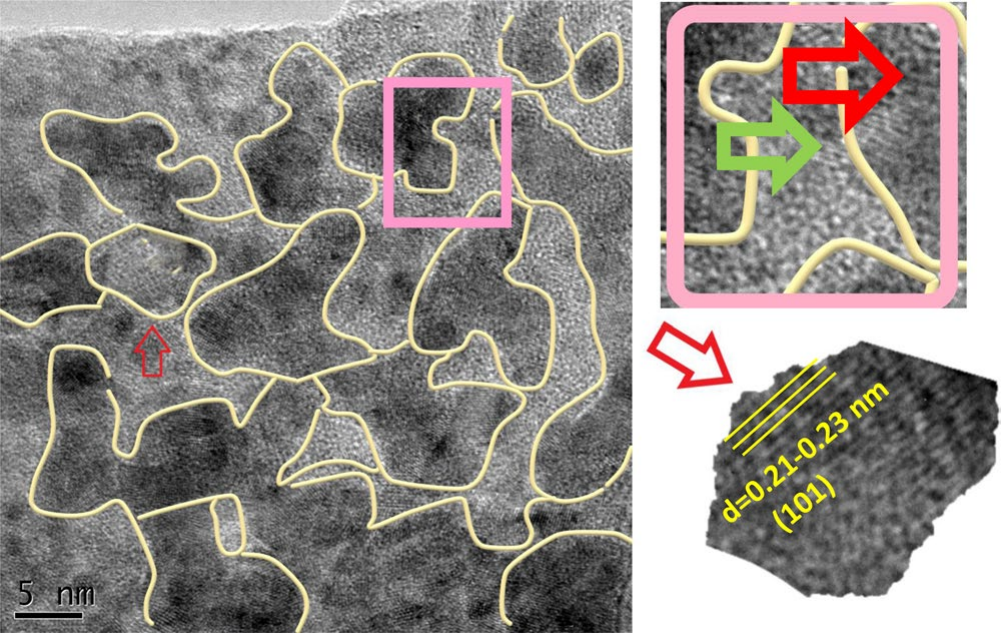
(HR)TEM images of the mechanically separated solid of the operated Ni foam at 10.0 V with different magnifications. The crystalline areas are showed in yellow boundaries. Red and green arrows show crystalline and amorphous areas, respectively. The HRTEM image indicates a crystal lattice spacing of 0.21–0.23 nm, corresponding to the (101) plane of Ni(II) hydroxide (ref.: 00-001-1047).

High-resolution transmission electron microscopy (HRTEM) images of the mechanically separated solid on the surface of the operated electrode at 10.0 V showed crystalline areas (ca. 5–10 nm) (Fig. 7). The HRTEM images also indicated a crystal lattice spacing of 0.21–0.23 nm, corresponding to the (101) plane of Ni(II) hydroxide (ref.: 00,001). (111) plane was also observed in the XRD pattern (Fig. S6).

SEM images of the foam as an anode electrode after amperometry at 1.46 V in KOH (pH ≈ 13) in a two-compartment electrochemical cell for 2 hours, showed nanoparticles (ca. 70–100 nm) (Figs. 6f–h, S7 and S8). The ratio of ions on the surface of nickel foam in Ni:Fe is 4.7:1.0. Thus, relatively large amounts of Fe are on the surface of the electrode.

XRD showed no patterns related to Fe compound on the surface of the Ni foam after amperometry at 1.46 V in KOH (pH ≈ 13) in a two-compartment electrochemical cell for 2 hours (Fig. S9). However, the patterns for the separated iron compounds indicated Fe(OH)_3_ (Fig. S10) (Ref. code: 00–022–0346; crystal system: Cubic; a (Å): 8.3700;b (Å): 8.3700; c (Å): 8.3700; α (°): 90.0000; β (°): 90.0000; γ(°): 90.0000; volume of cell: 586.38) and FeOOH (Ref. code: 01-076-2301; crystal system: Orthorhombic; a (Å): 3.8700; b (Å): 12.4000; c (Å): 3.0600; α (°): 90.0000; β (°): 90.0000; γ(°): 90.0000; volume of cell: 586.38 Å^3^). Only a trace amount of Fe could significantly activate Ni oxide for OER (Fig. 1). Thus, it is concluded that the separated phases such as Fe(OH)_3_ and FeOOH are not necessary for oxidizing water.

Oxygen-evolving catalysts are usually powders placed onto conductive substrates by binders. These binders decrease the contact between the electrolytes, electrode, and the catalyst and results in a decrease in the electric conductivity. The stability of these electrodes also is low because of the catalyst separation from the electrodes, especially under high current densities and harsh oxygen evolution. Using adding Fe salt to Ni oxide/Ni interface needs no binder and related limitations for the binder.

### OER of K_2_FeO_4_ and Ni_2_O_3_-based compounds under chemical conditions

**Figure 8:**
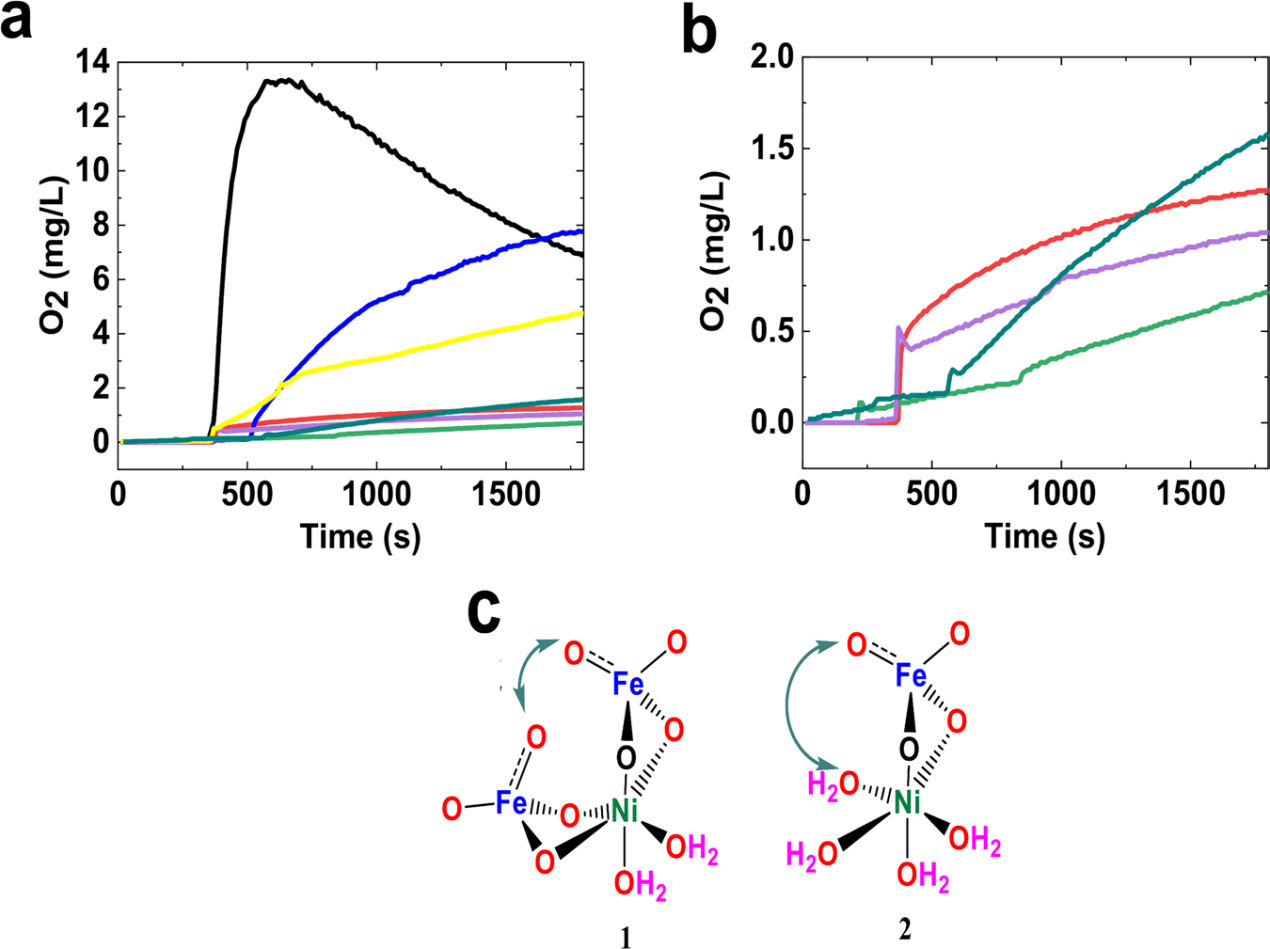
Oxygen evolution vs. time plot for Ni and Fe compounds at pH = 11 (Black: [Ni(NO_3_)_2_]: 2.76 mM, [K_2_FeO_4_]: 2.83 mM; blue: [K_2_FeO_4_]: 2.83 mM, [Ni_x_Fe_y_OOH]:36.2 mg; yellow: [Ni_2_O_3_]: 100 mg, [K_2_FeO_4_]: 2.85 mM; pink: [Ni_2_O_3_]: 200 mg; Fe(ClO_4_)_3_.5H_2_O: 1.50 mM, pH: 11; dark green: [Ni_2_O_3_]: 10 mg; [Fe(ClO_4_)_3_.5H_2_O]: 1.50 mM; light red: K_2_FeO_4_]: 2.83 mM and soluble Ni(II); total volume of solution 45 ml (**a,b**: different scales). Two mechanisms for the activation of [FeO_4_]^2−^ in the presence of Ni(II) ions (**c**).

In the next step, OER in the presence of K_2_FeO_4_ and Ni_2_O_3_ compounds are investigated under chemical conditions (Fig. 8). A few important questions in this regard are:

1. Whether OER of Ni_2_O_3_ is affected by the Fe(III) salt or not?
2. If OER of K_2_FeO_4_ is affected by the Ni(II) salt or not?
3. If OER occurs in the presence of both Ni_2_O_3_ and K_2_FeO_4_?

The answers to these questions are critical for understanding the mechanism of OER by Fe-Ni oxide. After the deaeration of a KOH solution (1.0 mM), Ni_2_O_3_ was added, but no clear oxygen evolution was observed (Fig. 8). In addition to this reaction, as shown by the electrochemical method, Ni(III) oxide in the absence of Fe ions or potential could not oxidize water. It was demonstrated that Ni(III) in the β-NiOOH-like phase has no activity. Though, it was reported that after the oxidation of Ni(III) to Ni(IV), the activity toward OER would be observed^30^.

In the next step, after the deaeration of Ni_2_O_3_ in KOH (1.0 mM), the Fe(III) ions was added. Again no detectable OER was observed. This shows that Fe(III) ions cannot activate Ni_2_O_3_ for OER. Regarding the Pourbaix diagram, it is also unlikely that Ni_2_O_3_ could oxidize Fe(III) ions (Fig. 8).

In contrast to Ni_2_O_3_, K_2_FeO_4_ is not stable, and OER is observed in the presence of this compound in a KOH solution (1.0 mM) (Fig. 8). Adding Ni(II) ions or nickel hydroxide significantly increased OER of a K_2_FeO_4_ solution in a KOH solution (1.0 mM). It displays that OER could occur by the mechanism of activation of K_2_FeO_4_ using Ni ions (Fig. 8c)^31^.

Another mechanism could be the formation of an Fe-Ni oxide by incorporation of Fe ions in Ni oxide. This Fe-Ni oxide could be oxidized by K_2_FeO_4_ in the next state and can oxidize water in the next step. To recognize the activation of K_2_FeO_4_ toward OER on the surface of Ni oxide, a saturated Ni(II) solution was prepared, and the decomposition of K_2_FeO_4_ was investigated in the absence of Ni oxide, but in the presence of trace but soluble Ni(II). In the presence of a K_2_FeO_4_ solution, adding a trace amount of Ni(II) ions causes no increase in OER.

In yet another step, an Fe/Ni hydroxide was synthesized, and OER of the compound was investigated in the presence of K_2_FeO_4_. Compared to soluble Ni(II) ions or Ni_2_O_3_, large amounts of oxygen evolution was detected in the presence of Fe/Ni hydroxide or Ni(II) hydroxide and K_2_FeO_4_. These experiments show that:

(i) Ni_2_O_3_ is relatively stable at alkaline conditions.
(ii) Ni_2_O_3_ is relatively stable at alkaline conditions and in the presence of Fe(III).
(iii) K_2_FeO_4_ is more active toward OER than Ni_2_O_3_.
(iv) Significant OER is observed in the presence of K_2_FeO_4_ and Ni(II) hydroxide.

Why Ni(II) hydroxide and not Ni_2_O_3_ significantly induces the decomposition of K_2_FeO_4_ could be related to the presence of labile Ni(II) ions on the surface of Ni(II) hydroxide, which contrary to an inert Ni(III), quickly form an intermediate such as **1** shown in Fig. 8. As the research on OER by Ni/Fe oxides are focused on the electrochemical issues^32–38^, these chemical experiments could be a road map to new findings.

## Conclusions

In the study, OER by Ni oxide in the presence of Fe ions under electrochemical conditions was investigated. [FeO_4_]^2−^ was used as a new, soluble in KOH, and colorful with negative charge Fe salt for investigating the effect of Fe on OER by nickel oxide. A Ni foam was operated at 10.0 V, and then the Ni oxide/Ni interface was used for the electrochemical investigation.

In the absence of the Fe salt and the presence of the impurity in KOH or electrochemical cell, an increase in OER was displayed during the consecutive CVs. The Ni oxidation peak shifted from 1.43 V to 1.46 V. After adding the Fe salt, a significant change is observed in OER, and the Ni oxidation peak is observed at 1.50 V.

SWV indicated the clear peaks for Ni(II)/(III) oxidation before and after adding the Fe salt. In the absence of the Fe salt in KOH (pH ≈ 13), a peak at 1.37 V was observed, which corresponded to Ni(II)/Ni(III) oxidation. After 50 consecutive CVs, the peak shifted to 1.39 V, which was attributable to the effect of Fe impurity. After adding the Fe salt, the peak was observed at 1.42 V. The effect of different scan rates on OER, Ni oxidation and reduction peaks were also studied. Both oxidation and reduction peaks for Ni in the presence and absence of Fe changed linearly by (scan rate)^1/2^. The oxidation peaks shifted to higher potentials, and reduction peaks shifted to the lower potential in the presence of Fe ions. Adding large amounts of the Fe salt had no significantly stronger effect on OER compared to adding a small amount of the Fe salt. SEM images of the foam as an anode electrode (amperometry at 1.46 V in KOH (pH ≈ 13)), after adding the Fe salt, showed nanoparticles on the surface of the electrode (ca. 70–100 nm). In the next step, OER of [FeO_4_]^2−^ and Ni_2_O_3_ under chemical conditions was investigated. The experiments showed that K_2_FeO_4_, compared to Ni_2_O_3_, is more active toward OER. Significant OER is observed in the presence of K_2_FeO_4_ and Ni(II) hydroxide.

## Methods

See Supplementary Information

## Supplementary information

Supplementary information.

Supplementary video.
