# Supplementary material for: Oxygen-evolution reaction by nickel/nickel oxide interface in the presence of ferrate(VI)
Source: Sci Rep. 2020 May 29;10:8757. doi: 10.1038/s41598-020-65674-x (PMC7260238; doi:10.1038/s41598-020-65674-x)
Supplement: Supplementary file 1 — Supplementary information. [file 41598_2020_65674_MOESM1_ESM.docx]

**Supplementary Information (SI)**

**Oxygen-evolution reaction by nickel/nickel oxide interface in the presence of ferrate(VI)**

Mohammad Saleh Ali Akbari^a^ Robabeh Bagheri,^b^ Zhenlun Song,^b^ and Mohammad Mahdi Najafpour^a, c,d*^

^a^Department of Chemistry, Institute for Advanced Studies in Basic Sciences (IASBS), Zanjan, 45137-66731, Iran

^b^Key Laboratory of Marine Materials and Related Technologies, Zhejiang Key Laboratory of Marine Materials and Protective Technologies, Ningbo Institute of Materials Technology and Engineering, Chinese Academy of Sciences, Ningbo 315201, China

^c^Center of Climate Change and Global Warming, Institute for Advanced Studies in Basic Sciences (IASBS), Zanjan, 45137-66731, Iran

^d^Research Center for Basic Sciences & Modern Technologies (RBST), Institute for Advanced Studies in Basic Sciences (IASBS), Zanjan, 45137-66731, Iran

*Corresponding author; Phone: (+98) 24 3315 3201; E-mail: [mmnajafpour@iasbs.ac.ir](mailto:mmnajafpour@iasbs.ac.ir)

**Experimental Section**

**Materials**

All reagents and solvents were obtained from commercial sources and used without further purification. K_2_FeO_4_, Ni(NO_3_)_2_.6H_2_O, and fluorine-tin oxide coated glass (FTO) were purchased from Sigma-Aldrich Company. KOH (Fe: ≤0.001%) was purchased from Merck Company.

**Ni(OH)_2_**

Ni(OH)_2_ (Figure S11) was synthesized by the reaction of Ni(NO_3_)_2_.6H_2_O (1.0 mM, 20 mL) and KOH (1.0 M, 20 mL)). The green precipitation was washed with water (three times, 10 mL) and dried at 60 ⁰C.

**Fe/Ni(OH)_2_**

Ni(OH)_2_ (20 mg) was dispersed in a Fe(ClO_4_)_3_.6H_2_O (1.0 Mm, 10 mL) under stirring (250 rpm) for one hour. The precipitation was washed with water (three times, 10 mL) and dried at 60 ⁰C.

**Methods**

SEM was carried out using an LEO 1430VP microscope. The X-ray powder diffraction patterns were recorded with a Bruker D8 Advance (Germany) diffractometer (CuK_α_ radiation). TEM was carried out with an FEI Tecnai G^2^ F20 transmission electron microscope (TF20 200 kV).

**Electrochemistry**

Electrochemical experiments were performed using an EmStat^3+^ from PalmSens (Netherlands). Cyclic voltammetry studies were carried out with a conventional three-electrode set-up, in which Ni foam, Hg|HgO, and a platinum foil served as working, the reference and the auxiliary electrodes, respectively. All potentials in this project were reported vs. Hg|HgO. The distance between two opposite sides of the electrode was measured by a digital caliper MarCal 16ER model (Mahr, Germany). For Fe free experiment, a polypropylene container was used. H_2_SO_4_ (6 M) in the polypropylene container was stirred for one hour and then carefully was washed by water.


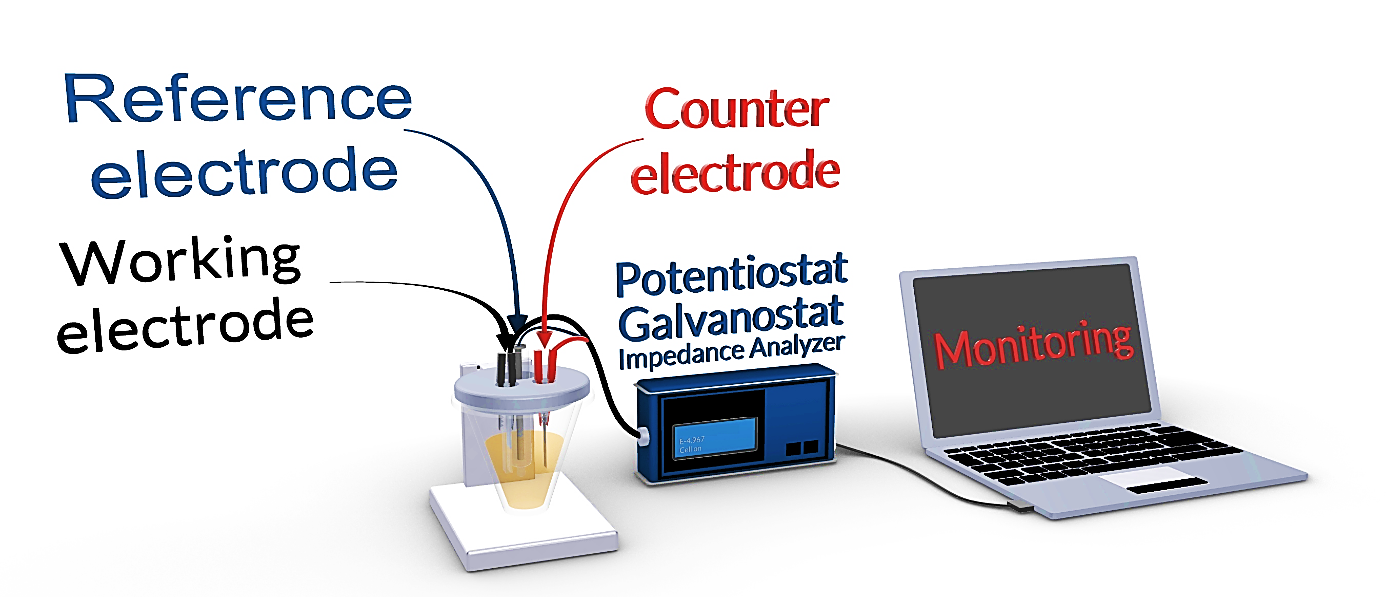


Scheme S1 Set-up for the electrochemical experiments. A similar set-up with salt bridge (KCl, 3.0 M) was used for two-cell set-up.

Figure S1 250 consecutive CVs of Ni foam without operating at 10.0 V (scan rate: 100 mV; KOH (pH≈ 13)) in the absence and presence of Fe salt. After the 10^th^ CV, Fe was added (a). The current density at 1.0 V for the consecutive CVs in the image a (b).


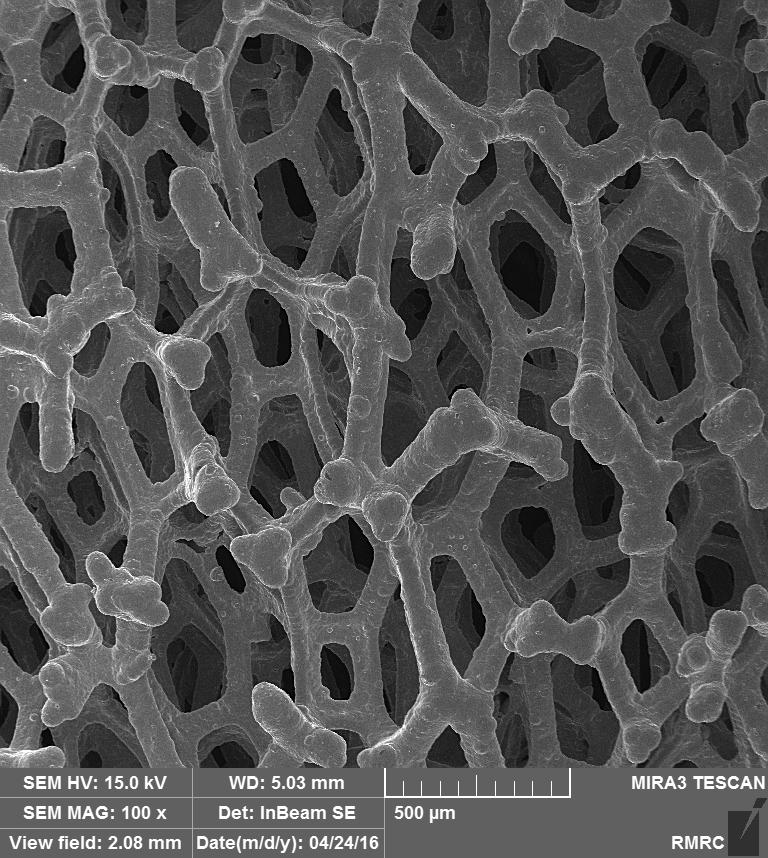

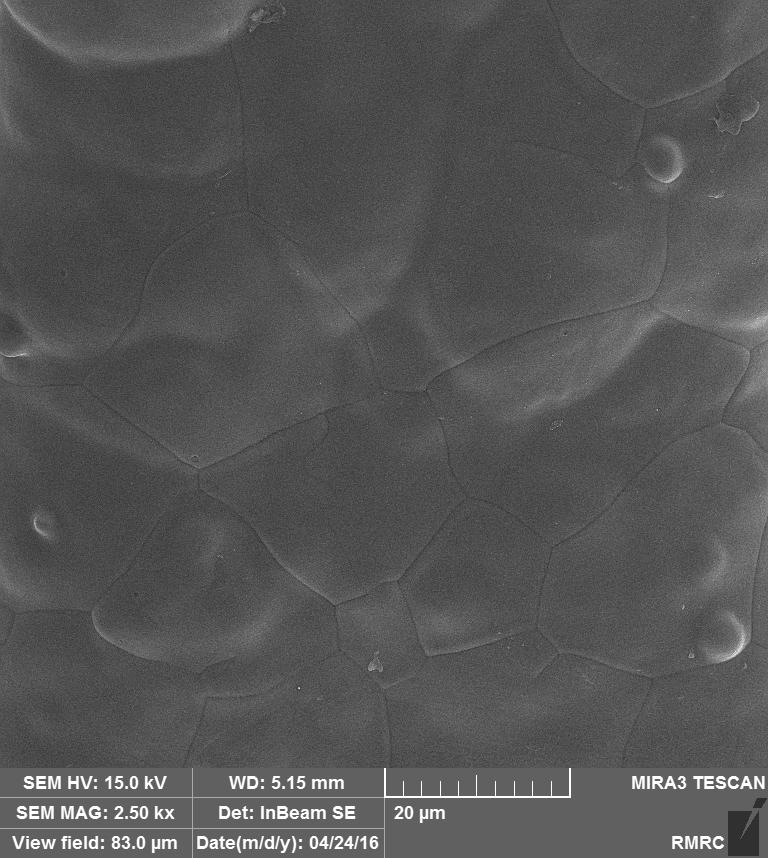


**c**

**d**

**b**

**a**


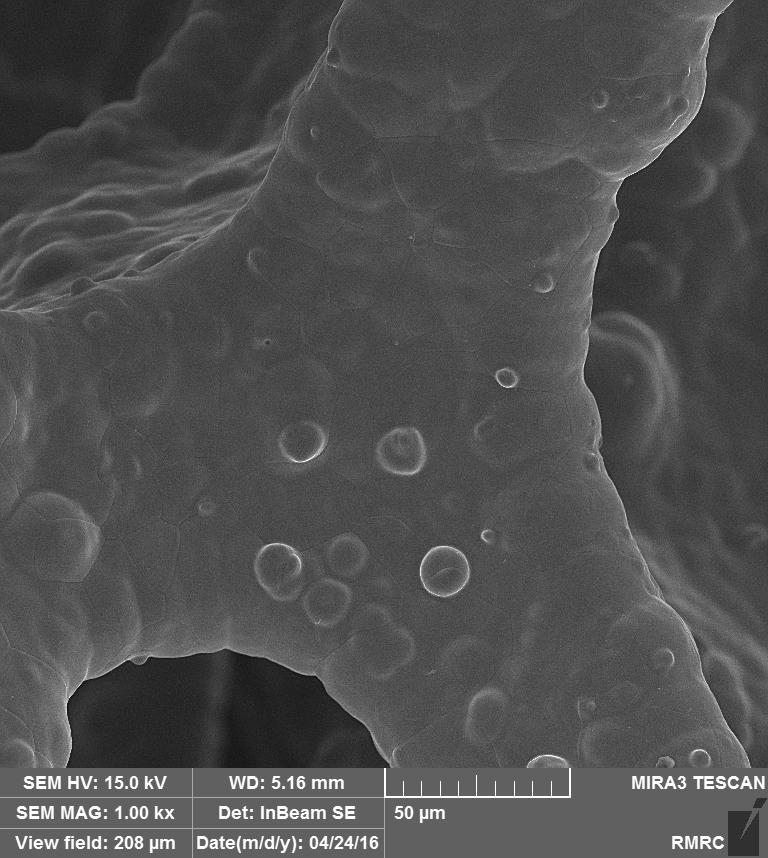

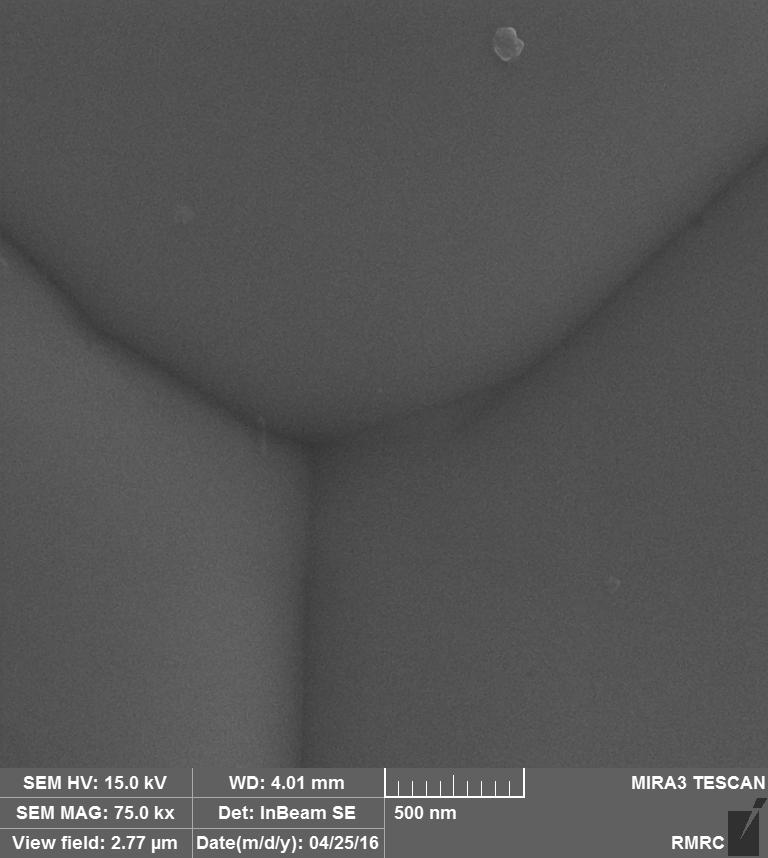


Figure S2 SEM images of a fresh Ni foam with different magnifications (a-d).


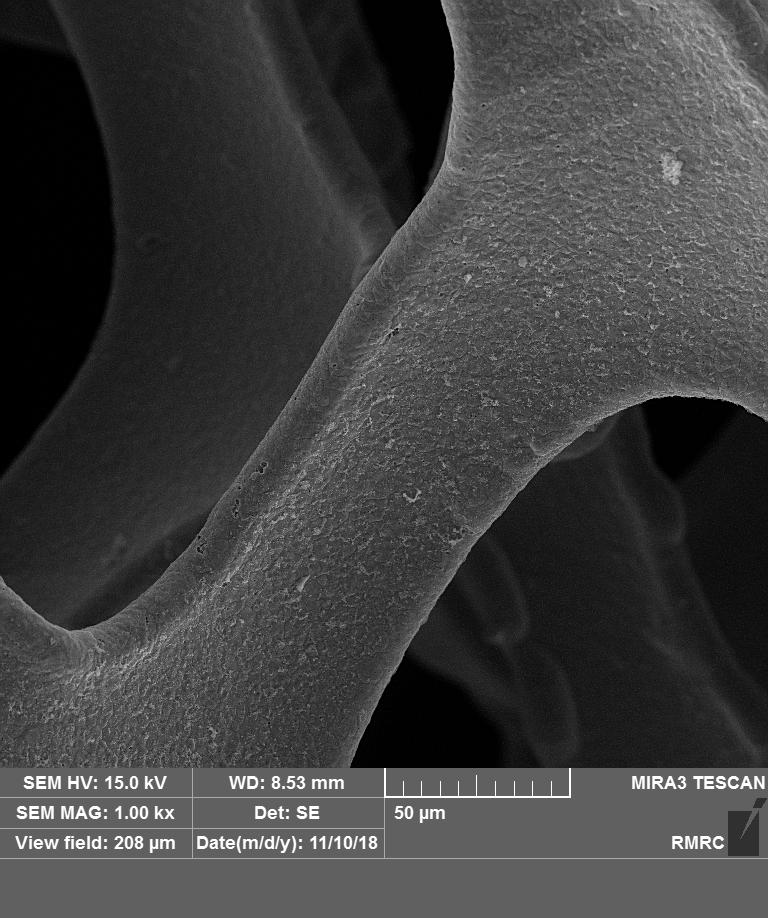

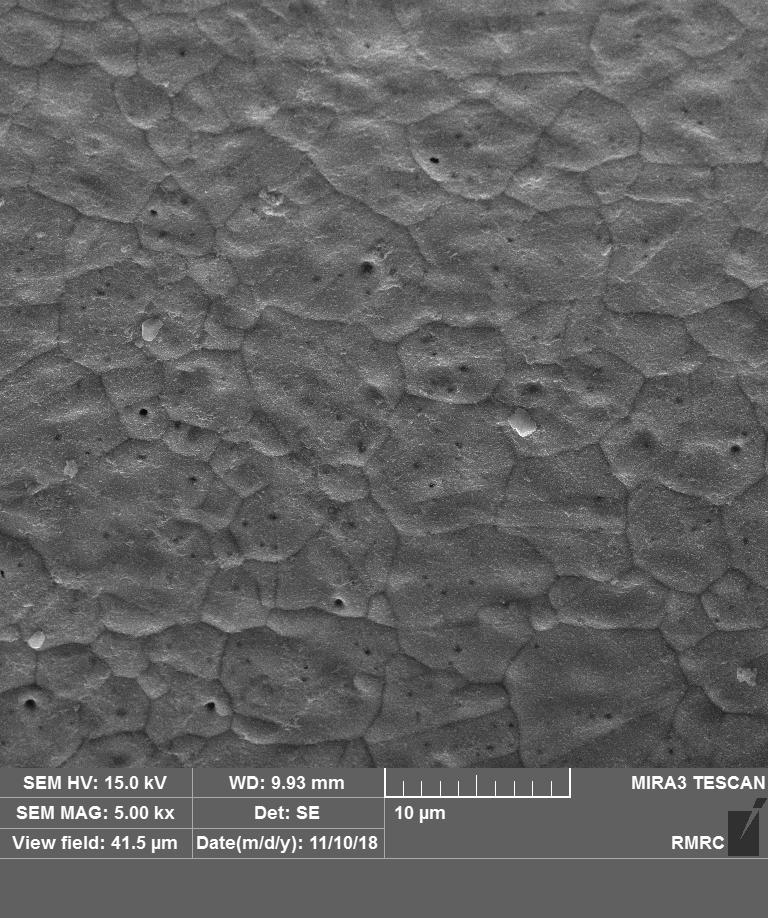

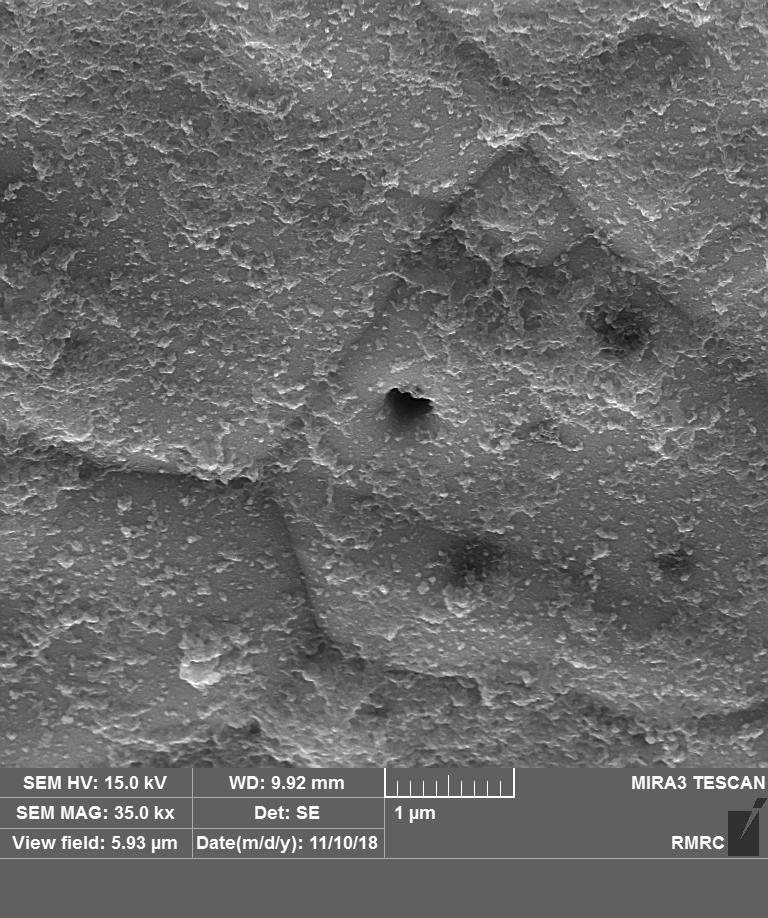

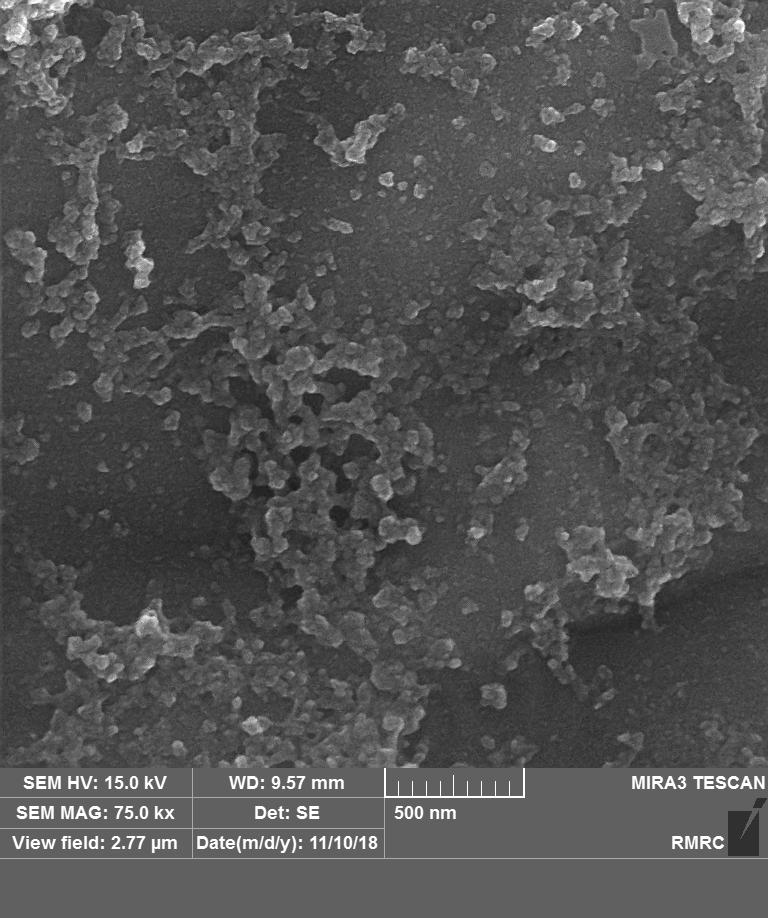


**a**

**b**

**d**

**c**

Figure S3 SEM images of the operated Ni foam at 10.0 V with different magnifications (a-d).

Fe

K


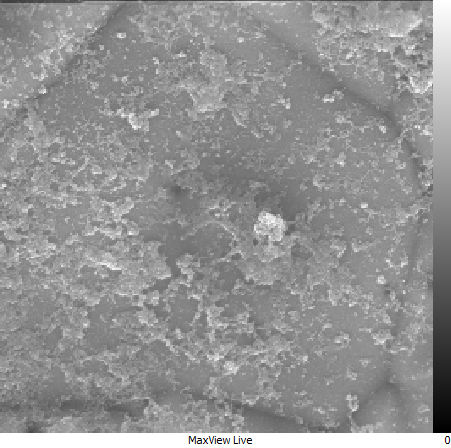

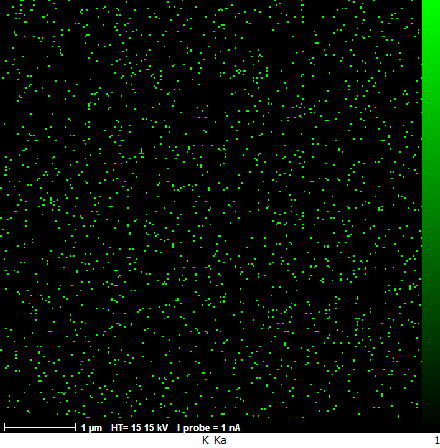

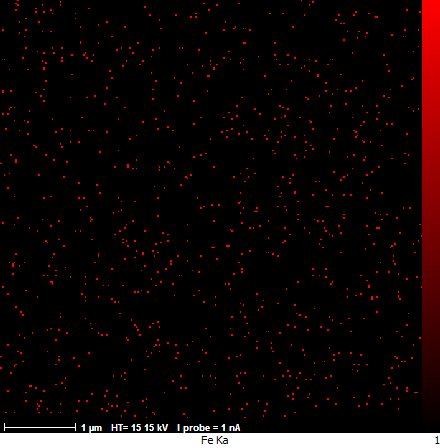

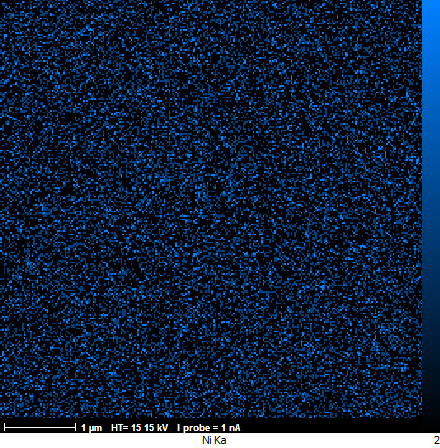

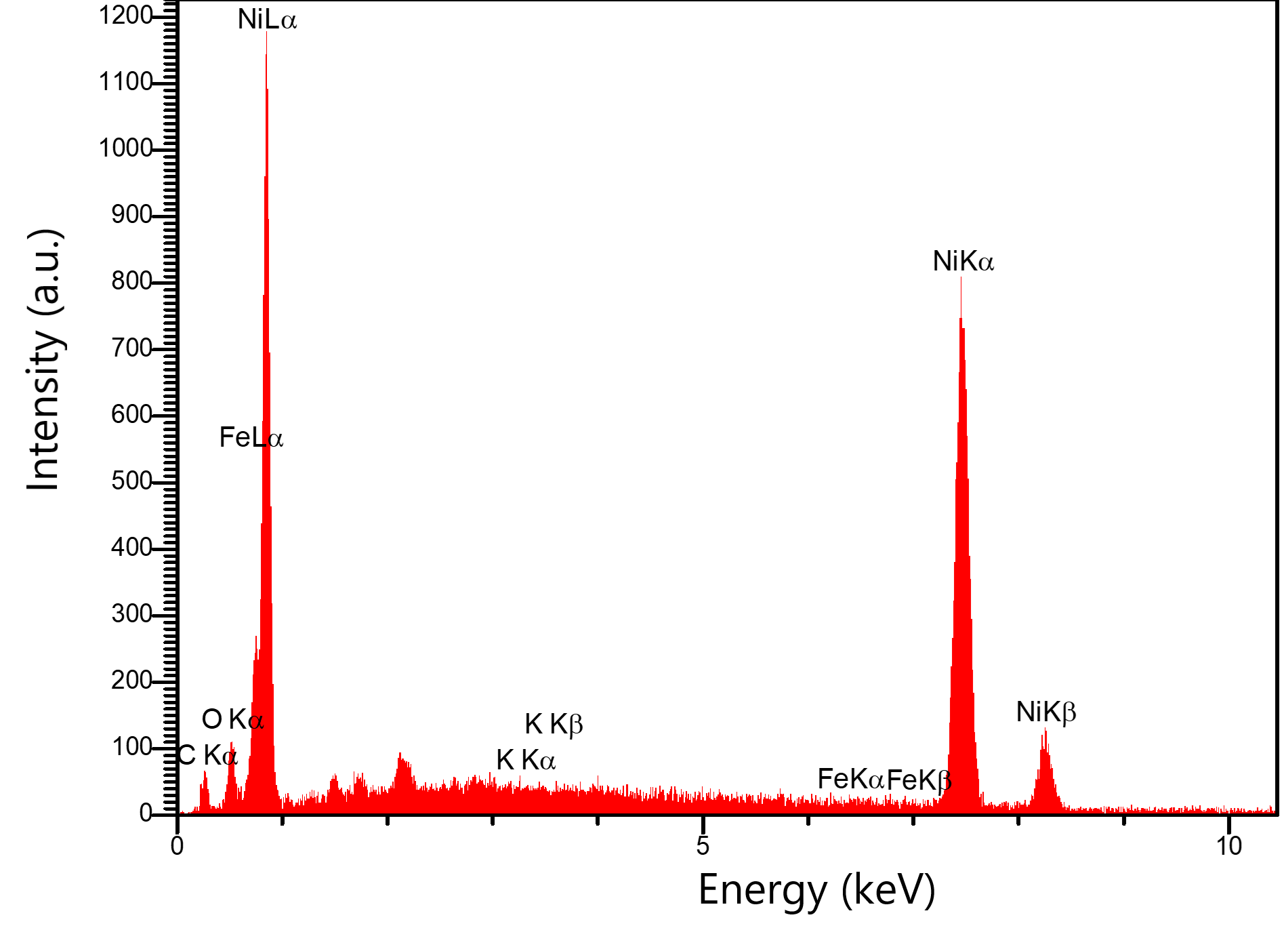


Ni

Figure S4 EDX-Mapping images and EDX spectrum of the operated Ni foam at 10.0 V with different magnifications.


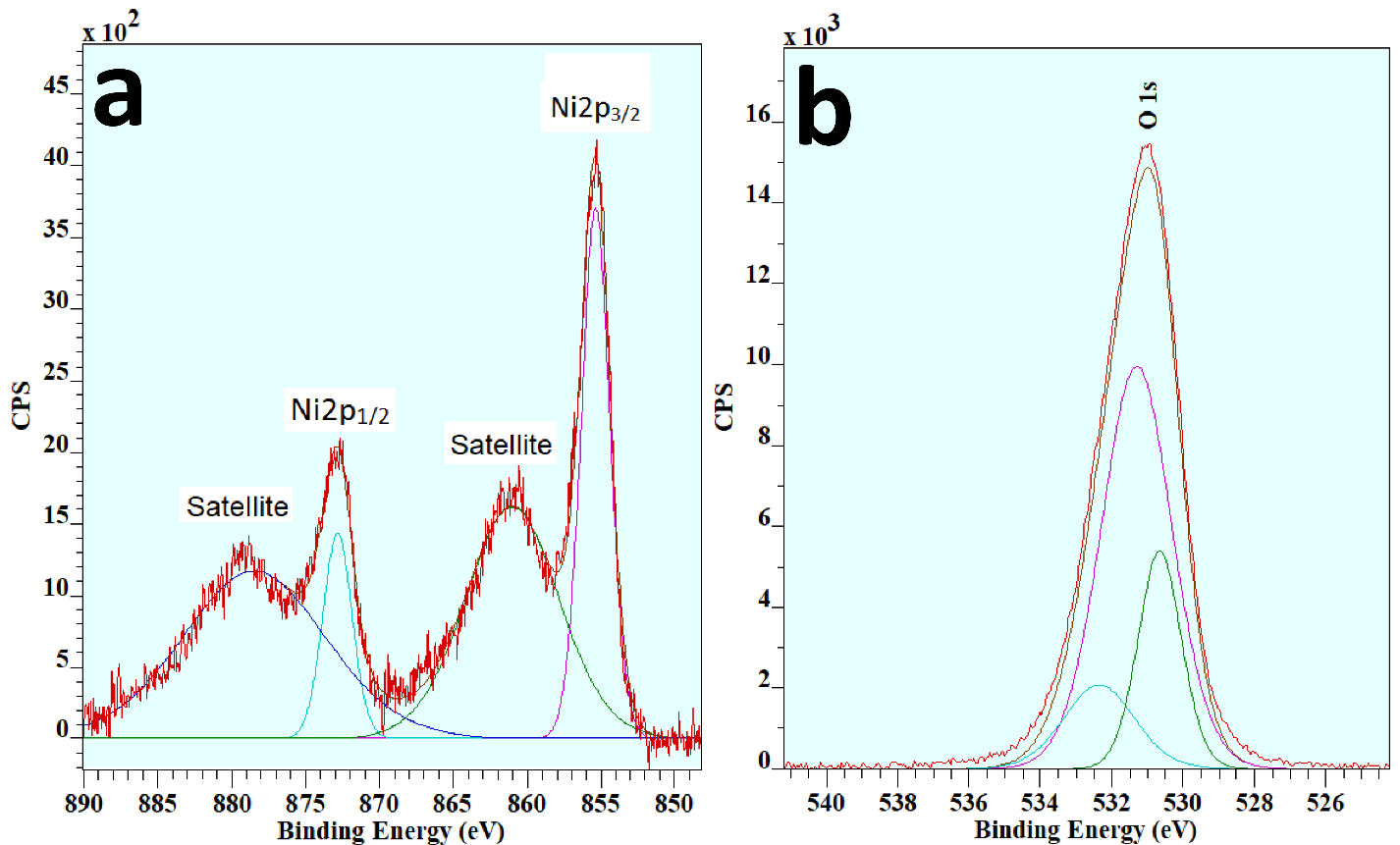


Figure S5 XPS spectra for Ni (a) and O (b) on the surface of the operated Ni foam at 10.0 V.

Figure S6 XRD patterns of a fresh Ni foam (blue) and the operated Ni foam at 10.0 V (red). The small peaks are related to Ni(II) hydroxide (black, ref.: 00-001-1047).


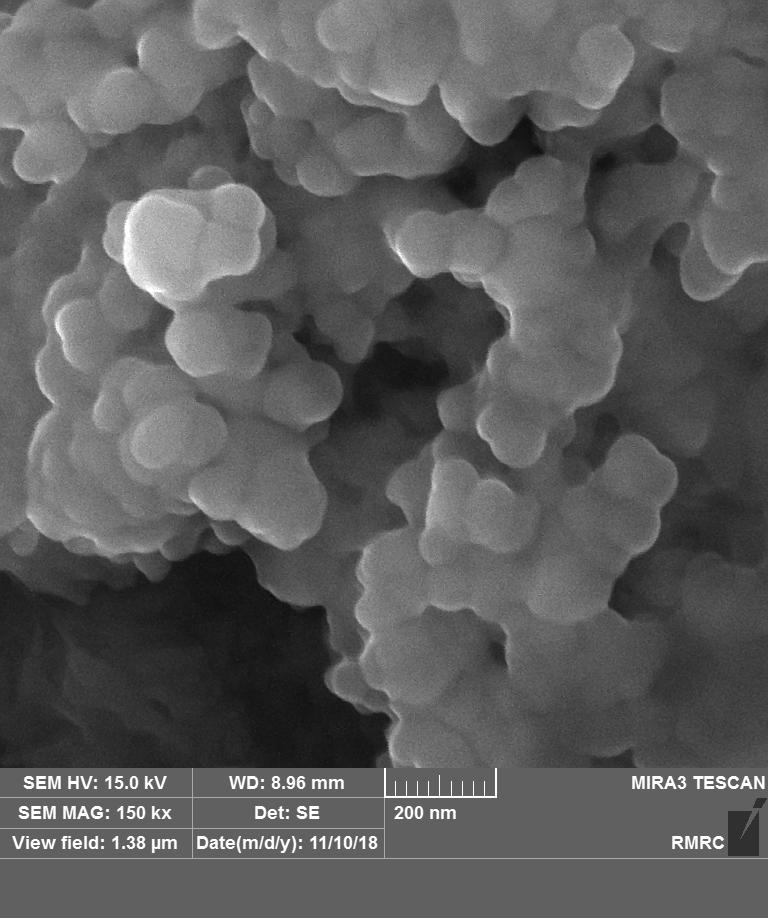

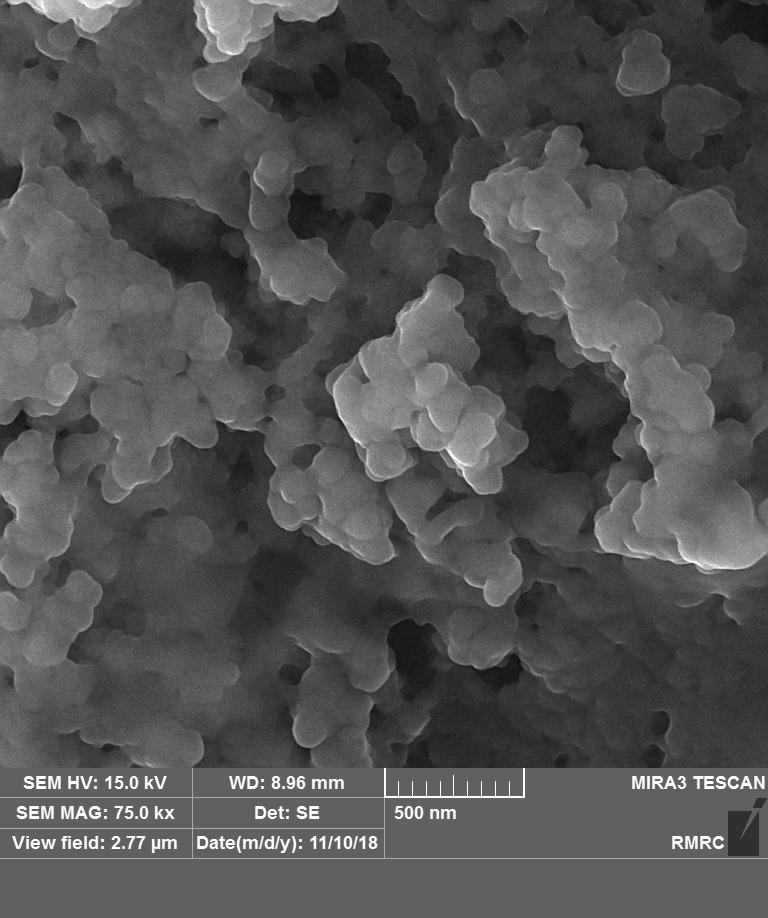

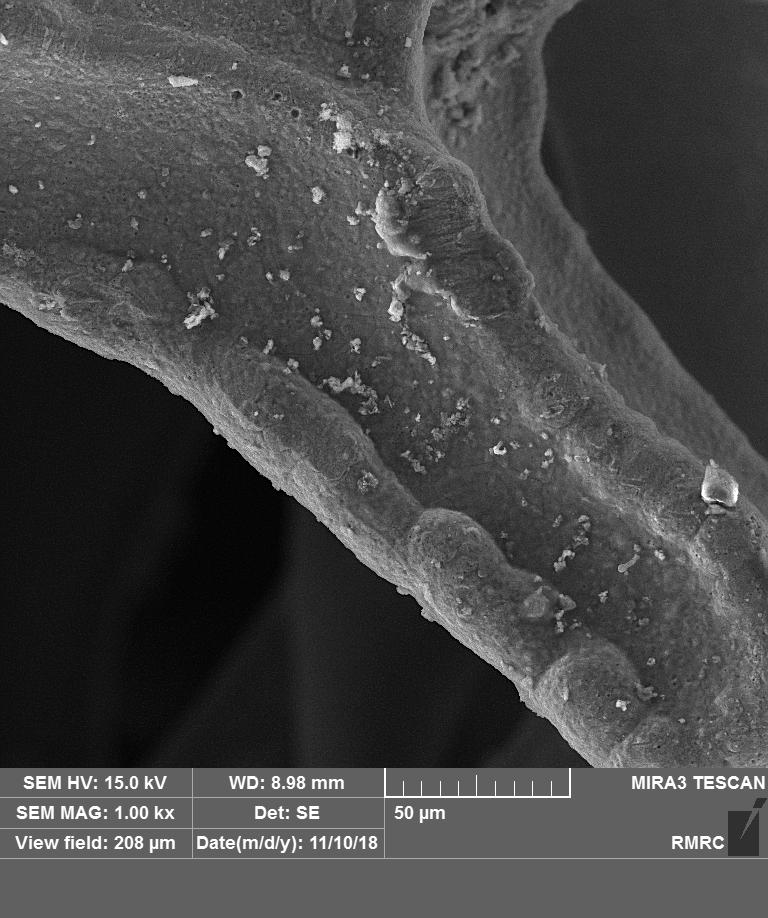

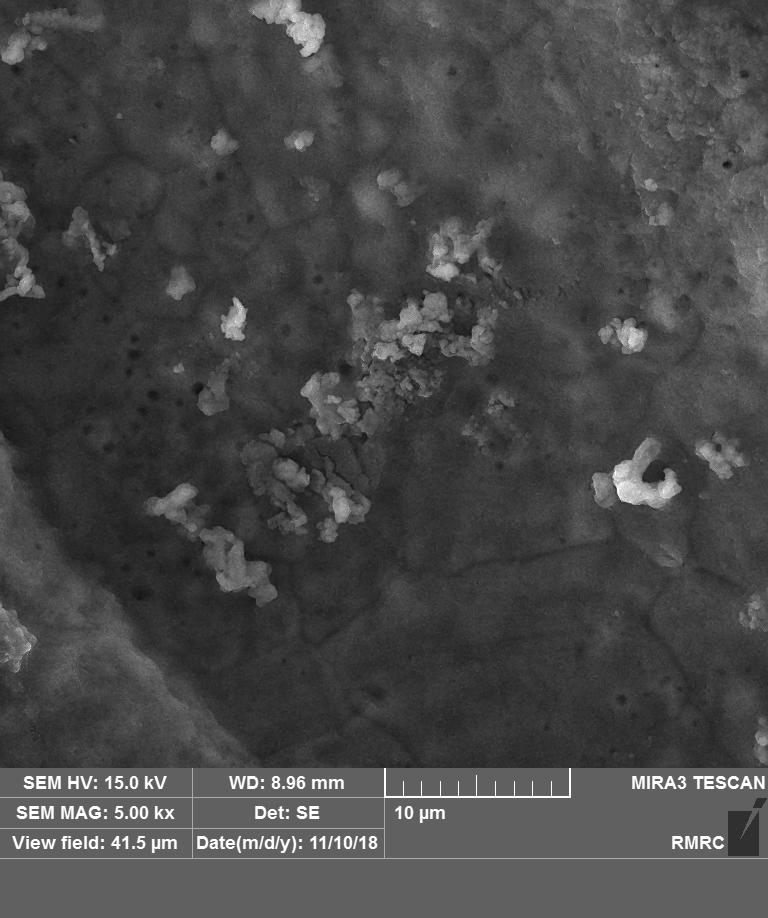


**d**

**c**

**b**

**a**

Figure S7 SEM images of the anodic Ni foam in the presence of iron with different magnifications **(a-d).** Amperometry at 0.80 V in KOH (pH≈ 13) in a two-compartment electrochemical cell for 2 hours.


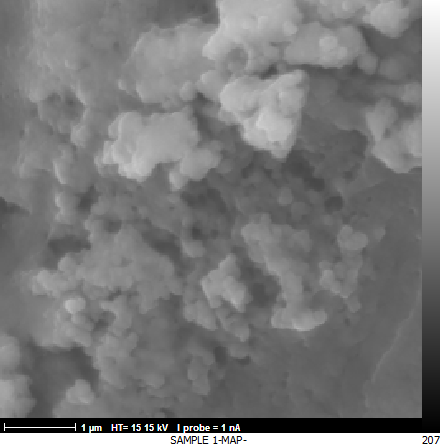

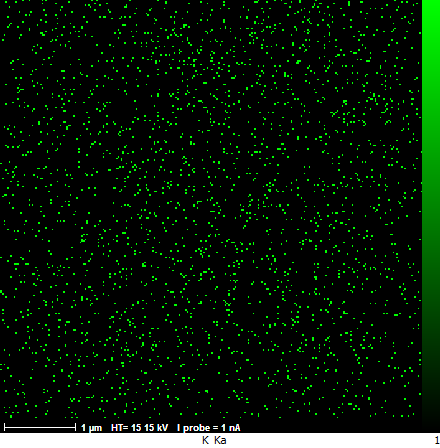

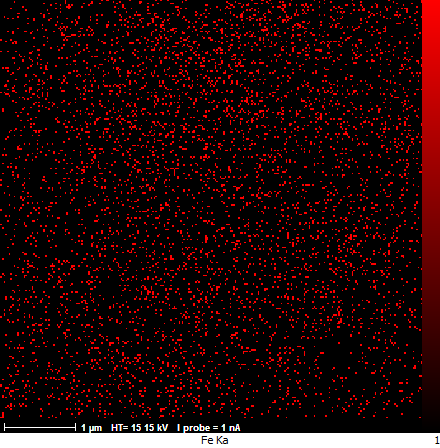

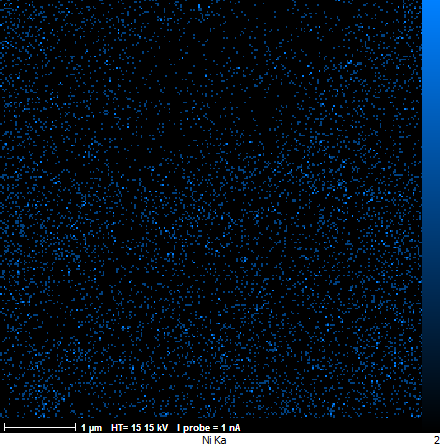

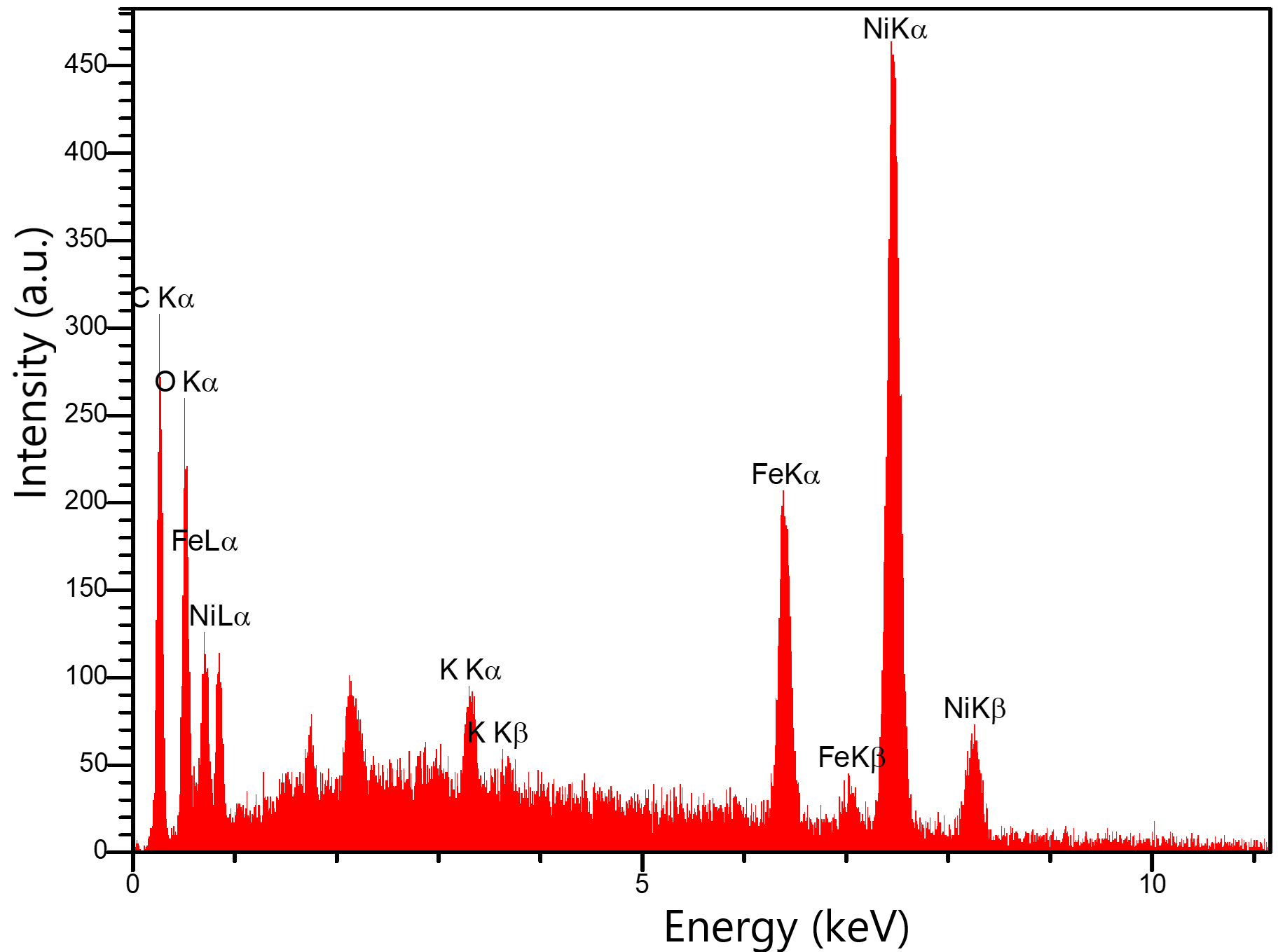


Ni

K

Fe

Figure S8 EDX-Mapping images and EDX spectrum of the anodic Ni foam. Amperometry at 0.80 V in KOH (pH≈ 13) in a two-compartment electrochemical cell for two hours.

Figure S9 XRD pattern of Ni foam in the presence of the Fe salt (final concentration: 6.3 mM) (orange) after amperometry at 1.46 V in KOH (pH≈ 13) in a two-compartment electrochemical cell for 2 hours. *Related peaks to metallic nickel.





Figure S10 XRD pattern of the solid dispersed in solution (red) in the presence of the Ni foam in the presence of the Fe salt (final concentration: 6.3 mM) after amperometry at 1.46 V in KOH (pH≈ 13) in a two-compartment electrochemical cell for 2 hours. Fe(OH)_3_ (Ref. code: 00-022-0346; green) and FeOOH (Ref. code: 01-076-2301; blue).

Figure S11 XRD patterns for the synthesized Ni(II) hydroxide (ref.: 00-001-1047).
